# An Overview of the Potential Therapeutic Applications of Essential Oils

**DOI:** 10.3390/molecules26030628

**Published:** 2021-01-26

**Authors:** Mariam Nasser Aljaafari, Asma Obaid AlAli, Laila Baqais, Maream Alqubaisy, Mudhi AlAli, Aidin Molouki, Janna Ong-Abdullah, Aisha Abushelaibi, Kok-Song Lai, Swee-Hua Erin Lim

**Affiliations:** 1 Health Sciences Division, Abu Dhabi Women’s College, Higher Colleges of Technology, 41012 Abu Dhabi, UAE; H00349760@hct.ac.ae (M.N.A.); H00323776@hct.ac.ae (A.O.A.); H00307981@hct.ac.ae (L.B.); H00349801@hct.ac.ae (M.A.); H00349412@hct.ac.ae (M.A.); lkoksong@hct.ac.ae (K.-S.L.); 2 Department of Avian Disease Research and Diagnostic, Razi Vaccine and Serum Research Institute, Agricultural Research Education and Extension Organization (AREEO), Karaj 31585-854, Iran; a.molouki@rvsri.ac.ir; 3 Department of Cell and Molecular Biology, Faculty of Biotechnology and Biomolecular Sciences, Universiti Putra Malaysia, Serdang, 43400 Selangor, Malaysia; janna@upm.edu.my; 4 Dubai Colleges, Higher Colleges of Technology, 17258 Dubai, UAE; aabushelaibi@hct.ac.ae

**Keywords:** essential oils, antimicrobial activity, oregano, thymol, carvacrol, cinnamon bark, synergistic activity, genomics, proteomics

## Abstract

The emergence of antimicrobial resistance (AMR) has urged researchers to explore therapeutic alternatives, one of which includes the use of natural plant products such as essential oils (EO). In fact, EO obtained from clove, oregano, thymus, cinnamon bark, rosemary, eucalyptus, and lavender have been shown to present significant inhibitory effects on bacteria, fungi, and viruses; many studies have been done to measure EO efficacy against microorganisms. The strategy of combinatory effects via conventional and non-conventional methods revealed that the combined effects of EO–EO or EO–antibiotic exhibit enhanced efficacy. This paper aims to review the antimicrobial effects of EO, modes of EO action (membrane disruption, efflux inhibition, increase membrane permeability, and decrease in intracellular ATP), and their compounds’ potential as effective agents against bacteria, fungi, and viruses. It is hoped that the integration of EO applications in this work can be used to consider EO for future clinical applications.

## 1. Introduction

Antimicrobial substances are secreted naturally in the microbial ecosystem as a survival mechanism. Antimicrobial-producing microorganisms, in order to avoid self-toxicity, commonly develop resistance genes to protect themselves from their own antimicrobials [1], while microorganisms faced with antimicrobials develop their own resistance in the process. This very same scenario is now replayed in the clinical setting whereby the application of antibiotics has caused the emergence of antimicrobial resistance (AMR) [2]. Some factors such as the inappropriate and incorrect prescription of antibiotics, their overuse in clinics and animal husbandry, in addition to poor sanitation of water in developing countries have exacerbated the phenomenon of antibiotic resistance [3,4,5,6,7,8,9,10]. Infections with multidrug resistant (MDR) microbes can be life-threatening as the clinical outcomes worsen [11,12]. This may also cause challenges for successful treatment [13].

Bacterial resistance to antibiotics can either be inherited or acquired via resistant genes [14]. Vertical gene transfer occurs from a parent cell while horizontal gene transfer of plasmids is acquired from other cells; the acquisition can also be due to a spontaneous recombination [9,15,16]. Due to the acquisition of these genes, bacteria are able to produce enzymes such as β-lactamase, carbapenemase, and nucleotidyltransferase (NTase) that act against the antibiotics, on the efflux pump on the bacterial cell membrane, or change the original target site of the antibiotic [17]. Fungi can also develop antifungal resistance. For example, *Candida*, an opportunistic yeast, can develop resistance to drugs when an antibacterial drug is consumed by the host. Despite the drug not being targeted to *Candida*, prolonged exposure can introduce *Candida* resistance to the drug [18]. Both *Aspergillus* and *Candida* species have shown resistance to antifungal drugs such as azole and fluconazole, which can be used to treat systemic fungal infections by inhibiting its growth [19,20]. Viruses have also shown antiviral resistance; for example, influenza virus has shown resistance against Oseltamivir, Amantadine, and Rimantadine. Resistance to Acyclovir by herpes simplex virus (HSV) has been detected in different countries [21].

In addressing the diminishing antibiotic pipeline, natural products such as essential oils (EO) have been studied as a promising alternative. Several cultures around the world used EO either for domestic use or for wound healing. In early 4500 BC, ancient Egyptians have used aromatic oils for different purposes; however, the first record of aromatic oils use was between 2000 and 3000 BC in Chinese and Indian medicine, which included the use of hundreds of substances for healing purposes. Between 400 and 500 BC, Greece has documented use of some EO such as thyme, peppermint, and saffron [22]. Furthermore, scientists in the 18th century have identified active components of medicinal plants that possess biological effects [22].

Compounds with functional groups in the EO were found to play a role in their reactivity against pathogenic microorganisms [23]. Therefore, the use of EO was taken as a new direction to treat microbial infections due to their antimicrobial, antifungal, and antiviral properties [24]. EO contain mixtures of volatile and concentrated organic compounds that are produced naturally in plants [25]. EO are produced in almost all parts of the plants such as flowers, fruits, leaves, stems, seeds, buds, twigs, bark, and stored in epidermal cells, glandular trichomes, secretary cells, cavities, or canals [26,27,28,29]. EO are plants’ secondary metabolites that play a significant role in plant reproduction, as they attract pollinators to contribute to distributing the seeds and pollens. However, some EO such as oil from *Cryptomeria japonica* also possess a repellent effect against arthropods and pests, which is a crucial part of the plant’s defense mechanism [30,31]. These EO and their constituents are well documented for antimicrobial potentials [32,33]. EO obtained from medicinal plants are reported to have more than 20–60 constituents of different concentrations and two to three main compounds, which are usually in high amounts (20–70%) [34].

Among the main components of EO are two distinctive chemical groups of bio-synthetic origin: terpenes and terpenoids. These compounds are usually responsible for antimicrobial action against disease-causing bacteria [35,36]. Terpenoids are diverse natural plant products that show various pharmacological properties due to their diverse structure and functions [37]. Terpenes are one of the most valuable classes of natural origin compounds that have economic value due to their use in different sectors such as in pharmaceuticals and food [38], with most of the common or medicinally important terpenes being monoterpenes and sesquiterpenes. Monoterpenes are found in about 90% of EO. These EO have a broad spectrum activity against bacterial infections due to their high content of oxygenated monoterpenes [39]. Terpenes are the hydrocarbons that consist of isoprene units with the general formula (C_5_H_8_)_n_ joined together by the head to tail rule—this meaning the fourth carbon atom of a single unit bonds with the first carbon atom of another unit to form a 1-4 linkage.

The role of EO is mainly in plant protection; apart from providing antimicrobial activities, they also provide protection against insects and herbivores [34]. According to the United States Food and Drug Administration (FDA) (2005), EO can be used safely, and its components can be used as additives in antimicrobial drugs to reduce the development of resistance against antibacterial, antifungal, and antiviral drugs [34,40,41]. Owing to the presence of various compounds, these EO have antimicrobial potential. This review summarizes EO effectivity on microorganisms and aims to extensively focus on the effectiveness of EO and their compounds against various microorganisms such as bacteria, fungi, and viruses. Moreover, we will discuss the potential of various EOs in inhibiting microbial growth in addition to identifying the mode of action of EO and synergistic activity. Finally, the genomic perspective of EO against bacteria and the proteomic technologies used to study the bacterial proteome when treated with EO will be discussed.

## 2. Essential Oil Research

### 2.1. AMR and Antimicrobial activity

Different compounds found in EO such as aldehydes, phenylpropanoid, and terpenes make EO effective against a wide range of pathogens [42,43], as the composition and the nature of each functional group gives each EO its reactivity [23,44]. Table 1 and Table 2 summarize the antimicrobial activity of a few selected and popular compounds in EO against different microorganisms.

#### 2.1.1. Antibacterial Activity of EO

Different EO exhibits different antimicrobial properties; this may include antibacterial activity. There are different mechanisms of action that cause the inhibition of microbial growth, which are still not fully known [51,65]. The antibacterial activity of EO refers to their ability to inhibit or inactivate bacterial growth [66,67]. Several studies have reported that some plants and EO such as clove, thyme, rosemary, oregano, cinnamon, and pimento showed potent inhibitory effects against various bacterial pathogens [68,69,70,71,72]. Studies suggests that phenolics found in EO such as eugenol, carvacrol, and thymol are mainly responsible for their antibacterial action against different bacteria such as *Staphylococcus aureus, Bacillus cereus, Streptococcus pneumoniae,* and *Escherichia coli;* phenolics pertains to the aromatic feature of EO [47,50,51,60].

In addition, it was determined that citronellol and carveol had a strong inhibitory effect over the growth of *E. coli* because these EO can interact with the cell wall components such as the membrane proteins, which will lead to disturbance in cell wall integrity [51]. Properties of certain compounds permit the ability of inhibiting the growth of most Gram-positive bacteria and a few Gram-negatives. This is explained by the outer structure of Gram-positive bacteria, which contains a thick peptidoglycan cell wall that allows penetration by hydrophobic compounds.

Moreover, EO from various sources such as oregano, basil, and rosemary from the mint family *Lamiaceae*, as well as parsley, coriander, and anise in the family of *Apiaceae* and cardamom from the *Zingiberaceae* family showed considerable antimicrobial activity against saprophytic microbes [73]. The result obtained from one study indicated the effectiveness of oregano EO against *Salmonella typhimurium, Yersinia enterocolitica,* and *E. coli* [73]. It was determined that oregano EO slowed the growth and lactic acid production of bacteria. Moreover, oregano EO when added to *S. typhimurium* significantly inhibited its growth [73]. In addition, oregano and lavender EO showed a bactericidal effect on *Klebsiella pneumoniae* with a Minimal Inhibitory Concentration (MIC) of 63,000 µg/mL [74]. Furthermore, cinnamon bark EO had been shown to cause oxidative stress to *K. pneumoniae*, leading to the loss of cell viability [75]. The results mentioned above showed both oregano and cinnamon bark EO possess great antibacterial benefit; in addition, many studies have investigated their efficacies as antimicrobials against several species.

Various studies have been conducted to investigate the antibacterial activity of plant EO. A study determined the effectiveness of pomegranate peel, grape seed cinnamon, oregano, and clove, but the highest antibacterial activity was shown by clove extract [76]. Moreover, *Echinophora platyloba* DC. EO showed a strong activity against bacteria, and *S. aureus* and *L. monocytogenes* were the most sensitive bacteria with MIC of about 6250 and 12,500 µg/mL [77]. Rosemary EO was tested against human tumor cells including SK-OV-3, HO-8910, and Bel-7402; it was shown to have a variety of activities, based on the MTT or 3-(4,5-dimethylthiazol-2-yl)-2,5-diphenyltetrazolium bromide assay. The EO was shown to possess antiproliferative cellular activity. The antioxidant activity was also assessed by measuring the inhibition of peroxidation percentage by the disc diffusion and resazurin plate assays. The results show that rosemary EO possesses antiproliferative, antioxidant activity, and antibacterial activity against different microorganisms tested [78,79].

A study that tested seven EO for their antimicrobial activity against resistant bacterial strains and fungi showed that oregano EO exhibited antibacterial activity against both *S. aureus* and *Streptococcus pyogenes* with the lowest MIC, which is 25 µg/mL, while other oils at higher concentrations were active against all microorganisms tested [80]. There were some EO that were more effective against Gram-positive bacteria while some were more effective against Gram-negative bacteria, and some EO were effective for both. *E. platyloba DC,* oregano EO, as well as the compounds with phenolic properties such as hydrophobicity have been found to inhibit Gram-positive bacteria while lavender, oregano EO, and citronellol, carveol compounds can inhibit more Gram-negative bacteria. Both Gram-positive and negative bacteria have been affected by citral, eugenol, carvacrol, and thymol EO compounds.

The demand and the usage of EO is currently gaining popularity not only for pharmaceutical uses but also in food, fragrance, and cosmetic industries. Their current use may well be expanded to eventually replace antibiotics, although their activity is still being established, as it is dependent on the chemical composition of the oil, which may function to inhibit bacterial growth in different ways, including causing cell wall disruption, metabolic pathway disruption, or reducing the cellular membrane potential [23].

#### 2.1.2. Antifungal Activity of EO

Fungal infections can be either superficial or invasive [81]. Treating human fungal infections mainly involves the use of oral tablets or topicals creams. It is also more difficult to apply treatment for fungal infections than bacterial, because human and fungal cells share the commonality of being eukaryotic. If the fungal treatment targets and acts against a common structure in eukaryotic cells, this may also lead to toxicity for the human cells, compromising host safety [81]. When developing an antifungal drug, it is important for pharmaceutical industries to target a structure that is found specifically only in fungal cells such as the chitin structure [81,82].

Accordingly, various EO and their individual compounds have been extensively tested against various fungal strains [83,84,85,86]. It has been found that EO exerted antifungal activity with roles in blocking cell communication, attenuating fungal growth, and inhibiting mycotoxin production [42,81,87].

The antifungal activity of multiple EO against *C. albicans* has been studied. Plants such as oregano, rosemary, and thymus showed a strong inhibitory effect with an MIC result ranging from 15.02 to 31.08 µg/mL [56,66,88]. Additionally, different *Ocimum* EO species have been tested against different *Candida* species, and their antifungal activity by the broth microdilution method have shown that *Ocimum micranthum* and *Ocimum selloi* are active against *Candida* species with MIC values of 312.5–1250 μg/mL [89]. Therefore, *Ocimum* EO can be used to treat infection with fungus such as *C. albicans* [89]. Due to an abundance of the phenolic constituents including carvacrol and thymol as major components in oregano and thyme, EO have shown significant inhibitory activity against fungal pathogens by breaking the fungal cell membrane [23]. In general, oregano has been found to affect both spore germination and destroy fungal cell membranes. In addition, it has been determined that the mycelial growth of three fungal *Aspergillus* species was inhibited by 90–100% due to the activity of cinnamon and clove EO [90]. A study has reported the fungicidal potentials of EO and pure constituents to hamper viable cell count, mycelia growth, and mycotoxin production by these fungi after treatment with clove, cinnamon, and oregano EO [42].

Main compounds such as terpenes, eugenol, farnesol, benzoquinone, menthol, and menthone show strong antifungal properties, especially against *C. albicans, Candida neoformans, Candida tropicalis, Candida glabrata*, and *Paracoccidioides brasiliensis* [45,54,56,58,91]. In general, EO can disrupt the chitin synthesis in fungal cell walls to causes abnormality in glycoproteins synthesis and mitochondrial structure, as well as in sporulation inhibition [81,82].

Furthermore, use of *Pinus sylvestris L.* (*Pinaceae*), *Origanum vulgare L.* (*Lamiaceae*), and *Thymus vulgaris L.* (*Lamiaceae*) EO and their main components to enhance itraconazole activity against azole susceptible/not-susceptible *Cryptococcus neoformans* strains has recently been reported, and the results have shown positive outcomes [92].

#### 2.1.3. Antiviral Activity of EO

Antivirals are medications that are utilized to treat viral infection via targeting the viral replication events resulting in an inhibition of viral replication [93]. The majority of effective antivirals against human immunodeficiency virus (HIV) and hepatitis B virus (HBV) has become ineffective due to antiviral resistance, although they have contributed to combating viral infections; several antivirals have also induced adverse reactions. As a result, many studies have been done to explore new antiviral treatments to these viruses [94,95,96,97]. In addition to the limited effective antiviral medications, alternative therapeutic substances such as EO have been explored, as several trials have shown that EO can possess significant antiviral activity against many RNA and DNA viruses by inhibiting viral multiplication [98].

Some EO compounds have shown an effective response against viruses through viricidal activity, preventing the viral replication and adsorption of viruses to host cells [99]. A study has proven the antiviral activity of several plants’ EO including *Zataria multiflora, Eucalyptus caesia, Artemisia kermanensis, Satureja hortensis L*., and rosemary against herpes simplex virus-1 (HSV-1) using the plaque reduction assay [100]. However, thymus EO was tested against HSV and influenza and showed no effective results [101]. In a different study, the β-caryophyllene, which is constituent in many EO, has been examined for its viricidal activity against dengue virus (DENV) proteins, and effective results were reported as it acted on inhibiting the replication of the DENV [102,103]. Compounds such as γ-terpinene and cuminyl-aldehyde have been found to have antiviral activity against two types of viruses; a significant inhibition was observed on HSV-1 (DNA virus), while a lesser inhibitory effect was observed on the parainfluenza virus type 3 (PI-3) (RNA virus) [97]. Moreover, ajwain EO, which was tested against Japanese encephalitis virus (JEV), was found to be effective with viral inhibition at 0.5 mg/mL of oil [104].

However, different EO will have different modes of action on viruses; in general, they act by targeting the nucleic acid polymerases. Generally, it has been shown that thymol and phenylpropanoids are compounds that have been responsible for the antiviral activity in HSV and JEV. EOs’ antiviral properties can be a promising alternative in the future, as more human trials must be conducted in order to provide more supportive evidence in terms of efficacy and safety.

### 2.2. Synergistic Activity in EO

The combinatory effect of EOs and their compounds with antibiotics is a new approach that is currently being explored. Combination therapy will result in three types of effects: synergistic, additive, and antagonistic [105,106]. Exploiting the synergistic combination of EO and antimicrobial agents have been suggested as one possible alternative strategy for combating antimicrobial resistance. According to a number of studies, several EO constituents have shown the ability to enhance conventional antimicrobial efficacy and potency when used in combination [105,106].

In a study, a combination of five EOs with seven antibiotics have been investigated; the combined effect of peppermint, cinnamon bark, and lavender EO with piperacillin, peppermint, and meropenem showed significant synergistic effects against various *E. coli* strains [25,107,108]. In a different study, the fractional inhibitory concentration (FIC) of EO was evaluated, and the data showed promising outcome against fungal and bacterial species [109,110]. Hence, when more than one EO were combined, a greater efficacy was achieved. Oregano and thyme EO combined showed synergistic effects against different fungal species studied with FIC ≤0.5, except for *Aspergillus niger*, which that displayed an additive effect with an FIC value of 0.75 ± 0.16. Moreover, a synergistic effect was found between carvacrol and thymol, which are compounds contained in thyme and oregano, respectively, to have a synergistic effect against *Penicillium spp*, *A. flavus,* and *Fusarium* species with FIC ≤ 0.5. A synergistic effect was also reported between carvacrol and thyme EO that showed an enhanced effectiveness against *S. typhimurium* [109,110].

Tea tree oil is known for its medicinal uses, mainly due to its antimicrobial effects [17,111]. Tea tree oil has been shown to react synergistically with tobramycin against *E.coli* ATCC 25922 and *S. aureus* ATCC 29213, with a mean post-antibiotic effect of 1.3 h against *E. coli* and 1.7 h against *S. aureus* [112]. This study showed that this combinatory therapy is effective against the Gram-positive and Gram-negative bacterial strains. In addition, tea tree oil in combination with peppermint oil showed a synergistic activity against *A. niger* with an FIC of 0.43 ± 0.06 [110]. Additional combinations of tea tree oil with different types of EO have been shown to give different synergistic and additive activities against fungal pathogens such as *Penicillium chrysogenum, A. flavus, Aspergillus parasiticus,* and *A. niger*. For instance, tea tree combined with cinnamon or eucalyptus showed additive activity in all fungal species with 0.5 ≤ FIC ≤ 1. If combined with thyme, the combination will give an additive activity with 0.5 ≤ FIC ≤ 1 against all species except *A. niger*. Moreover, the combination of oregano and tea tree has been shown to be additive against both *P. chrysogenum* and *A. niger* [110].

EO from *Salvia fruticosa, Salvia officinalis*, and *Salvia sclarea* were investigated using five *Staphylococcus epidermidis* strains that possess the *Tet(K)* efflux pump, which is involved in the tetracycline resistance mechanism [113]. The checkerboard method was used to evaluate the combined effect of the EO and tetracycline. Using quantitative RT-PCR, the largest decline of the tetracycline effluxes was detected from *S. epidermidis* cells treated with *S. fruticosa* EO. The mRNA level of *Tet(K)* gene was lowered by 2·2-fold, which indicated efflux pump inhibition. Findings demonstrated the synergistic potential of *Salvias’* EO combined with tetracycline, with lowered MICs values showing *S. fruticosa* EO to be the most effective of the three EO tested [113].

Furthermore, the synergistic antifungal, allelopathic, and antiproliferative potential of *S. officinalis l*. and thymus *vulgaris l.* EO have been investigated [114]. The results indicated that the tested EO alone as well as in combination had allelopathic effect, while the synergistic effect of *S. officinalis l.* and *T. vulgaris l*. EO in terms of fungal growth was found to be 0.06%. Furthermore, thyme and sage EO exhibited in vitro antiproliferative activity on melanoma cell lines A375 and B164A5 alone, as well as in combination [114].

The antifungal activity of "Mentha of Pancalieri" EO, either alone or in combination with azole drugs (fluconazole, itraconazole, ketoconazole) was assessed against a wide panel of yeast and dermatophyte clinical isolates [115]. The EO was analyzed by MIC in addition to GC-MS and minimum fungicidal concentration (MFC) parameters. The results suggested that the EO may act as a potential antifungal agent and could serve as a natural adjuvant for fungal infection treatment.

The antifungal effect of *Pelargonium graveolens* has also been tested in combination with fluconazole on *C. albicans* strains using the micro-broth dilution assay. The results suggest the synergistic reaction when combining *P. graveolens* with fluconazole with an FIC of 0.37, which shows that *P. graveolens* can enhance the efficiency of fluconazole treatment by 78.31% [116]. In addition, *Melissa officinalis* EO has been examined against avian influenza virus (AVI) [117]. The combination of *M. officinalis* EO and oseltamivir antiviral drug showed synergistic activity as the results showed a reduction in the viral genome copy number, which proves the inhibitory effect against AVI [117].

### 2.3. Mode of Action of EO Compounds on Pathogenic Bacteria

**Figure 1:**
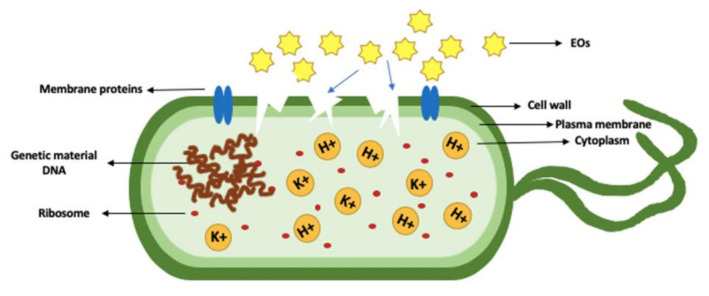
Membrane disruption in a bacterial cell caused by EO leading to the inhibition of bacterial growth. K+ is potassium ion and H+ is hydrogen ion.

EO chemical compounds possess different modes of action that can be used to inhibit or kill microbes (Figure 1) [23]. The mode of action of each EO compound may differ, as some act on the outer membrane of bacteria and some act on the membrane proteins’ efflux system. The hydrophobicity of EO enables them to penetrate the cell wall of bacteria, which subsequently disrupts the cell wall, causing increased permeability and the release of intracellular materials [51,118].

EO exhibit several antibacterial mechanisms that lead to the inhibition of bacterial growth; it has been proven through numerous studies that the membrane disruption phenomenon is one of the modes of action exhibited by EO’s constituents through targeting the cellular membrane as observed on the *L. monocytogenes* membrane upon applying oregano and thyme EO [119].

Bacterial cell membranes are composed of phospholipids and proteins. After the bacteria had been exposed to CBO, some proteins were lost from the outer membrane and some were lost from the plasma membrane of the bacteria [75]. These proteins that were lost play a role in energy generation; losing these proteins may disrupt the bacterial cell membrane and subsequent killing of the bacteria [75]. Moreover, the lavender EO (LVO) has been shown to cause oxidative stress to bacteria by oxidizing the outer membrane of the bacteria [120,121,122]. In fact, LVO and meropenem, in addition with reactive oxygen species (ROS), have resulted in bacterial membrane disruption.

The efflux pump is a protein found in the plasma membrane of the bacteria, and its major function is to prevent the entrance of toxic compounds into the cytoplasm [17,123]. This protein plays an important role in antibacterial resistance that helps bacteria to survive. A major mechanism of antimicrobial resistance is increasing the expression of efflux pumps in bacteria, which will subsequently result in lowering the antibiotic concentration in bacterial cells [65]. The efflux pump of bacteria works to protect the bacterial cell by pumping a large amount of antibiotics out of the cells [124]. Recently, some studies have found that EO are able to inhibit the efflux pump of the bacteria, and the compounds present in the EO have many targets on the bacterial cells [125]. Therefore, the inhibition of efflux pumps is a vital target for EO and their metabolites [126].

**Figure 2:**
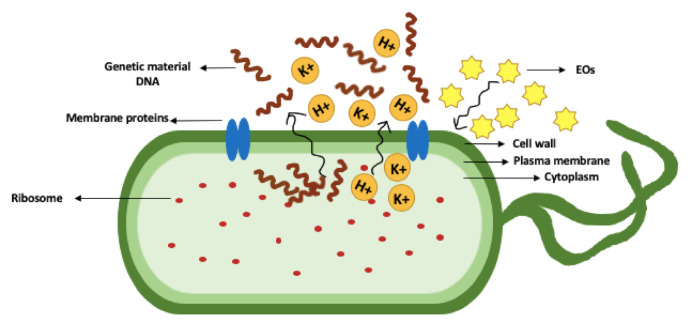
Increased membrane permeability and leakage of cell contents in a bacterial cell. K+ is potassium ion and H+ is hydrogen ion.

EO are hydrophobic in nature; thus, the hydrophobicity of the EO compounds causes an increase in the membrane permeability of bacteria, which may subsequently lead to the leakage of bacterial cell contents [44]. The leakage of the cell content includes ions such as potassium ion (K+) and hydrogen ion (H+), and also proteins and genetic material such as DNA (Figure 2) [127,128]. For example, the loss of cell content in *Bacillus subtilis* after exposure to *Origanum compactum* EO is an indication of the increased membrane permeability, which results in cell lysis [128].

**Figure 3:**
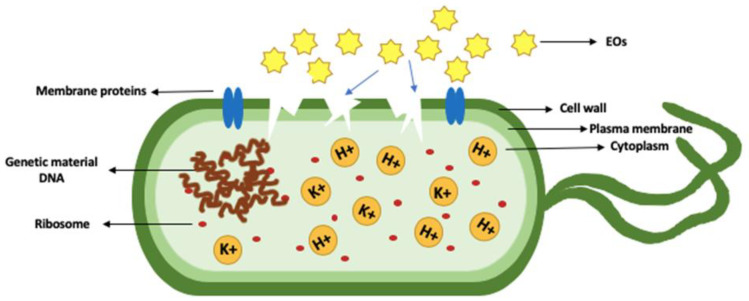
Modifications in ATP levels: (**A**) Normal ATP within the bacterial cell, (**B**) Decreased intracellular and increased extracellular ATP after exposing bacteria to EO.

ATP is an energy source found in all organisms, including bacteria, and it is essential for respiration and metabolic processes. ATP can be depleted by EO; the mustard EO caused the reduction of ATP intracellularly and increased extracellular ATP (Figure 3) in *E.coli* O157:H7 and *S. typhimurium* [44,129]. Furthermore, cinnamaldehyde and cinnamon oil decreased the intracellular ATP in *Mycobacterium avium* subspecies *paratuberculosis* (MAP) [130]. A high concentration of EO compound such as carvacrol can result in cell lysis by depleting intracellular ATP in *B. cereus* [131].

## 3. Recent Approaches

### 3.1. Genomics Perspective

There is a crucial need for the discovery of novel groups of antimicrobial agents to counteract the threat of MDR pathogens to reduce the rapid emergence of acquired resistance [132,133]. Therefore, it is important to know how EO operates at the genetic level and how they modulate microbial genes [134]. The availability of the complete genome sequence for several pathogenic microorganisms provides extremely useful information regarding potential drug targets and is a very useful resource in order to mine for novel antimicrobial drugs [135]. Using this approach, genome databases coupled with bioinformatics are used as tools for the transcriptional examination and recognition of the molecular basis. This will enable us to screen compounds/molecules as potential inhibitors of pathological targets and may also help us discover and optimize more effective next-generation antibiotics.

In regard to EO activity on bacterial genes, it has been found that rosemary and *Baccharis psiadioides* EO have shown a bacteriostatic effect that impacts the development and functions of bacterial cells of *L. monocytogenes* by upregulating and downregulating stress and virulence genes such as *actA*, and *hly*, thus reducing bacterial virulence [136]. In addition, by using scanning electron microscopy, qPCR, and a 96-well plate method, cinnamon oil has been found to have activity against the expression of *icaA* gene and biofilms of *S. epidermidis* [137]. Furthermore, confocal laser scanning microscopy (CLSM) has shown that cinnamaldehyde is able to kill the tested bacteria, thus indicating its effectiveness in biofilms [137]. All these findings prove that different EO can modulate gene expression by upregulating and downregulating stress and virulence genes of different microorganisms.

### 3.2. Proteomics Perspective

Amongst the recent approaches is the use of proteomic technologies for the effective analytical evaluations and modifications in protein profiles. Indeed, these methods are important tools to study the mechanisms of AMR in microbes by extensive analysis of the proteome [138]. They have been used to separate, identify, and quantify the different classes of EO components and will be discussed below.

Two-dimensional polyacrylamide gel electrophoresis (2D-PAGE) is known as the most used technique for separating and identifying proteins as it is able to evaluate thousands of different spots in a protein mixture [139]. It consisted of the isoelectric focusing (IEF) method in the first dimension, which separates the proteins according to the isoelectric focusing, and in the second dimension, separation is performed based on the molecular size by sodium dodecyl sulfate-polyacrylamide gel electrophoresis (SDS- PAGE) [140]. The basic principle of these techniques is to separate the complex protein mixture into two steps by placing the mixture in a gel and applying an electrical current. This technique is widely used in proteome analysis, bacterial pathogenesis, diseases research, and the purification of proteins [140].

Matrix-assisted laser desorption/ionization time-of-flight mass spectrometry (MALDI-TOF MS) is another emerging technology that has been proven to efficiently provide precise and reliable microorganisms identification results and has been employed in detecting antibiotic resistance as well [141]. The sample is mixed with an energy-absorbent matrix, and a laser beam is used to ionize the samples. Then, the molecules are charged, and consequently, the time of ion flight may differ according to their mass-to-charge ratio (m/z) value [142].

Understanding the protein profiles helps researchers find effective interactions of EO components and drugs with their targets, which are mostly proteins in nature. Over the years, MALDI-TOF MS has proved its ability to detect and quantify changes that occur in the proteomes of the bacterial cells after the exposure to EO compounds using different proteomic techniques. For example, it has been used for the identification of stress response in *E. coli* upon exposure to EO components [143]. Overall, MALDI-TOF MS has been more cost-effective, reliable, and rapid compared to other diagnostic tools [144].

To identify the mechanism of action of thymol EO, 2D-PAGE followed by MALDI-TOF methods was employed to analyze the protein profile in order to detect the induced stress reaction caused by exposing *Salmonella enterica* serovar Thompson to sublethal concentration (0.01%). The study demonstrated that different significant effects occurred including proteins alteration, increase, and reduction. Proteins such as GroEL and DnaK were overexpressed in the treated cells as a reaction due to treatment by EO. However, the thioredoxin-1 protein was downregulated due to thymol action. In conclusion, utilizing proteomics approaches to observe the effects was helpful to provide evidence of thymol bactericidal ability and show its particular targets [145].

Other powerful and precise analytical tools that are used for the assessment of the bacterial metabolome include gas chromatography-mass spectrometry (GC-MS), liquid chromatography coupled to tandem mass spectrometry (LC-MS), nuclear magnetic resonance (NMR) spectroscopy, and microarray. LC-MS/MS is a technique used for the quantification of proteins that is highly selective and sensitive [146]. LC-MS/MS may be used to analyze the effect of EO on the proteome of bacteria by comparing the proteome of the cells that are treated with particular EO against untreated cells [120].

On the other hand, the combination of GC and MS creates a powerful analytical tool that separates compounds by subjecting them to heat, which allows the determination of their chemical structure. Once the volatilization of the compound occurs, the quantification and qualitative identification of the compound can be recorded. These techniques enable researchers to identify major constituents and their concentrations present in EO.

Furthermore, NMR instruments allow the molecular structure of a material to be analyzed by observing and measuring the interaction of nuclear spins under a powerful magnetic field. The method allows cellular components to be easily examined and recognized from their characteristic chemical shifts. In general, intra and extra-cellular metabolomic studies using the methods mentioned in this section have some basic benefits such as the provision of important functional genomics, characterization of the strains, their metabolic engineering, and ways of cellular communications [147]. Bacteria that are resistant to beta-lactam antibiotics were studied using proteomic analysis [148]. The study revealed an increase in several proteins related to antibacterial resistance mechanisms such as the outer membrane protein (To1C) that is involved in the efflux pump system [148].

## 4. Limitations in Essential Oils Research

Although extensive studies have been conducted to explore the antimicrobial potential of EO, the results of several studies are not entirely in agreement in terms of the EO efficiency killing microbes or mitigating their stability at various environmental conditions, such as in the presence of salts. There are also limited studies on the relationship between the structure of EO compounds correlated to antimicrobial activity [149]. However, the mechanisms by which the EO function are not fully understood in detail. Moreover, there have been reports of the negative impact of excessive doses of EO on human health [150].

The variation in results might be due to disparity in EO, level of various impurities in samples, type of microbes, and experimental conditions. Furthermore, various studies have indicated slightly variable definitions of MIC and Minimum Bactericidal Concentrations (MBCs). The main issue is that EO may not be easily tested by routine techniques such as disc diffusion and MICs may not be easily tested in the traditional way that powdered materials are tested, because EO undergo volatile evaporation from the discs may provide inaccurate results. Consequently, there is a great variation in results and reproducibility of the results, and it is often very difficult to draw useful and applied results from various research studies.

Despite the fact that EO are highly useful alternative antimicrobial agents, especially against food-borne microbes, there is a considerable lack of information regarding their toxicity profiles. Moreover, the likelihood of EO effect on the stress tolerance of microbes is limited. Though generally regarded as safe, natural products and EO are not completely safe due to the diversity of metabolites in them when used as antimicrobial agents [151]. The large amount of secondary metabolites may have synergistic or antagonistic properties when used in non-standardized form. Thus, there is a need to optimize the analysis of a range of EO and compounds more clearly for the effective yet safe use of EO for human beings. The formation of residues in biological systems and allergic reactions associated with the use of EO are also among other challenges regarding their use as antimicrobial agents [152].

Stability is another issue associated with the use of EO due to their heat-labile nature. EO are generally volatile at normal temperature and need to be stored in cool and dry environments. Exposure to elevated temperatures and humidity might cause their decomposition and hence efficacy as antimicrobial agents to be reduced. For instance, cinnamaldehyde was reported to be degraded at elevated temperatures and form benzaldehyde at 60^◦^C, but when combined with eugenol or cinnamon leaf oil, it is stable even at high temperatures (200 °C for 30 min). More recently, encapsulation tools were reported to significantly improve stability issues and reduce excessive oxidation, loss of quantity, change in aroma, and interactions with other chemicals [153,154]. Nevertheless, more detailed research studies are required regarding the potential use of encapsulated EO as efficient antimicrobial agents.

Although a plethora of EO are available, only a few have obtained approval as preservatives in food. This is because certain food components drastically reduce the antimicrobial efficiency of EO. Consequently, the development of more effective and validated food model systems that have close resemblance with food components is desirable. This will predict the effect of food on the antimicrobial potentials of EO and will help in the optimization of EO as food preservatives. Subsequently, it is very important to evaluate the antimicrobial mechanisms of various food combinations with EO for optimized and reliable use of these oils as preservatives. As discussed earlier, the combination of various secondary metabolites is sometimes required for the adequate antimicrobial efficacy of EO. Some studies have reported that crude extracts and crude oils display a better antimicrobial spectrum when compared with pure compounds [23]. This might be attributed to the synergy between various metabolites or the combined antimicrobial effect of various components.

Natural products must be carefully evaluated for the antimicrobial activity of their purified EO before describing them as effective or ineffective antimicrobial agents. EO can be used in combination with common herbs such as spices, which will improve their preservative capacity for various foods including fish, sauces, meat, and soups. As mentioned earlier, EO alone may offer only a mild antimicrobial spectrum that leads to an ineffective organoleptic profile for users. As a consequence, research on the combined synergistic effect of EO and herbs may be designed in the future for a more effective and optimized utilization of these oils as preservatives while preserving their aroma and spicy taste.

## 5. Future Strategies and Prospects

EO reactivity depends on their functional groups; they can increase permeability of the pathogen cell membrane and cause the leaking of intracellular components, which negatively affects the cell metabolism [23,155]. Moreover, EO are important for reducing the antimicrobial resistance; more effort is needed to do long-term studies to determine the EO effectiveness in vivo by conducting clinical trials in order to discover its full potential, the needed dose, and determine if any side effects emerge [23,99]. EO can be effective by either the absorption, ingestion, or inhalation through lungs of its volatile compounds [25,156,157]. These oils are effective and used in raw and processed food preservation, perfumery, and in alternative medicine due to their antimicrobial and antioxidant activities for burn healing, and malarial infection [25,158,159]. EO are also shown to give synergistic activity when used with antibiotics and thought to be effective to treat antimicrobial resistance, and in the public point of view, it is more accepted due to its traditional use [23,160,161]. Previous discoveries of EO against antimicrobials still did not explain exactly the EO mechanism of action and their chemical nature, and this makes the effectiveness and action of EO against antimicrobials unclear and in need of improvement [23,162].

In the future, research studies should be done to determine the exact mechanism of action that is specific for each EO and the synergistic mechanisms between its components [34]. Further extensive studies might be designed to evaluate or predict the adoptive behavior of microbes to the EO after chronic or sub-chronic use. As reported in previous studies, *B. cereus* have become less susceptible to the antimicrobial effect of carvacrol subsequent to culturing at low concentrations of the compound [163]. Meanwhile, a significant enhancement in the sensitivity of the same bacteria to the action of EO was observed after alteration in the phospholipids and fatty acids composition, which alter the fluidity and passive permeability of the bacterial cell membrane. Additionally, the effect of EO on the bacterial membrane proteins and phospholipids and their mechanisms is not fully understood yet. A few EO have been shown to have low toxicity to the human body; however, other EOs may affect human cells negatively and might be considered unsafe to be used. Thus, more studies are needed to check EO toxicity through many clinical phases [164,165].

Some researchers suggested that the antibacterial effect of EO should be checked in the lag phase of bacterial growth, which will help researchers understand the mechanisms and pathways involved in the development of EO as potential antimicrobial agents [34]. Nevertheless, with the emerging concept of green consumerism, it is expected that the use of EO and medicinal plants will increase, and further exploration will occur. The use of natural products-based drugs, nutraceuticals, herbs, and isolated pure compounds is tremendously increased in medicine, the food sector, cosmetics, and didactics. The use of modern analytical techniques can help mankind in the further development of evidence-based medicine with more efficacy and safety. Thus, new nanoencapsulation strategies and synergistic studies can help provide powerful information about this topic of interest in future [34,124].

Despite the fact that genomic technologies have provided us with tremendous opportunities to understand the biological drug targets, the problem of microbial-mediated infections is still a global health challenge. This is mainly due to the emergence of resistant genes and the indiscriminate use of antimicrobial agents. Another reason is that the pharmaceutical industries are in a rush to develop more drugs and are only getting derivatives of the original drugs. The discovery of novel compounds with new mechanisms of action is very limited. Thus, for the development of more effective therapeutic agents, experts from various disciplines are needed; these include genomics, structural biology, genetics, and bioinformatics. Moreover, the establishment of widespread epidemiological networks capable of reporting the emergence of new microbes and public awareness is imperative for effective mitigation.

## Figures and Tables

**Table 1:** Antimicrobial activity of some essential oils (EO) compounds against different microorganisms.

Antimicrobial Activity	EO	Main Compound	Structure	Microorganism	MIC/IC_50_	MBC/MFC	Reference
^a,b^Antibacterial activity	Peppermint and mint oils	Menthol	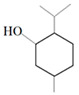	Methicillin-resistant *Staphylococcus aureus*—ATCC 33591 *Escherichia coli*—ATCC 10798 *Streptococcus mutans*—ATCC 25175 *Aggregatibacter actinomycetemcomitans*—ATCC 33384	1000 μg/mL >2500 μg/mL 1000 μg/mL 500 μg/mL	1000 μg/mL >2500 μg/mL 1000 μg/mL 1000 μg/mL	[45,46]
	Lemon and cinnamon oil	Linalool	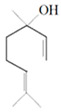	*S. aureus*—ATCC 25923 *E. coli*—ATCC O157:H7	5.0 μg/mL 6.0 μg/mL	5.5 μg/mL 6.0 μg/mL	[45,47,48]
	Clove oil	Eugenol	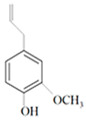	Methicillin-resistant *S. aureus* *E. coli*—S17 strain *E. coli*—ATCC 8739 *S. aureus*—ATCC 25923 *Bacillus cereus*—ATCC 14579 *Salmonella typhimurium*—ATCC 14028	1300 μg/mL 400 μg/mL 30 μg/mL 3 μg/mL 70 μg/mL 70 μg/mL	1.5 mg/mL 0.5 mg/mL Bacterial growth Bacterial growth Bacterial growth 0.06 mg/mL	[45,49,50,51,52,53]
	Ginger oil	Gingerols	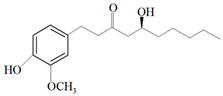 6-Gingerol 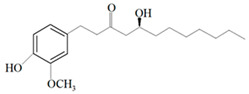 8-Gingerol 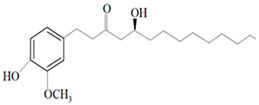 10-Gingerol	*Porphyromonas gingivalis*—ATCC 53978 *Porphyromonas endodontalis*—ATCC 35406 *Prevotella intermedis*—ATCC 25611	6–30 µg/mL 6–30 µg/mL 6–30 µg/mL	4–20 µg/mL 4–20 µg/mL 4–20 µg/mL	[54]
	Mustard oil	AITC	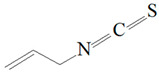	*S. aureus*—ATCC 29413 *Listeria monocytogenes*—(Scott A) *Salmonella enteritidis*—(PT 30)	500–1000 μg/mL 500–1000 μg/mL 500–1000 μg/mL	Not Available	[55]
^a,c^ Antifungal activity	Clove oil		Compound not specified in article	*Cladosporium cladosporioides*—air-borne *Chaetomium globosum*—air-borne *Aspergillus fumigatus*—air-borne	500 μg/mL 250 μg/mL 250 μg/mL	0.075% (*w*/*v*) 0.05% (*w*/*v*) 0.075% (*w*/*v*)	[56,57]
	Tea tree oil		Compound not specified in article	*Epidermophyton floccosum* *Microsporum canis* *Trichophyton rubrum* *Aspergillus niger* *Penicillium* spp. *Alternaria* spp. Fluconazole-Resistant *Candida albicans*—ATCC 10231	80–300 μg/mL 40–300 μg/mL 80–300 μg/mL 600–1200 μg/mL 300–600 μg/mL 160–1200 μg/mL 1250 μg/mL	0.12–0.25% (*v*/*v*) 0.06–0.25% (*v*/*v*) <0.03–0.25% (*v*/*v*) 2–8% (*v*/*v*) 0.5–2% (*v*/*v*) 0.06–2% (*v*/*v*) 0.25% (*v*/*v*)	[58]
	Arborvitae		Compound not specified in article	*C. globosum*—air-borne	100 μg/mL	0.025% (*w*/*v*)	[57]
	Oregano		Compound not specified in article	*A. fumigatus*—air-borne *C. cladosporioides*—air-borne *Alternaria alternata*—air-borne	250 μg/mL 100 μg/mL 100 μg/mL	0.075% (*w*/*v*) 0.075% (*w*/*v*) 0.05% (*w*/*v*)	[57]

^a^ MIC = Minimal Inhibitory Concentration. ^b^ MBC = Minimum Bactericidal Concentration. ^c^ MFC= Minimum Fungicidal Concentration.

**Table 2:** Antimicrobial activity of individual compounds from EO against different microorganisms.

Antimicrobial activity	Main Compound	Structure	Microorganism	MIC/IC_50_	MBC/MFC	Reference
^a,b^Antibacterial	Cinnamaldehyde	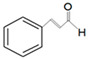	*Escherichia coli*—S17 strain Methicillin-resistant *S. aureus*	200 μg/mL 400 μg/mL	0.3 mg/mL 0.5 mg/mL	[50,53]
	Carvacrol	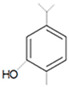	Methicillin-resistant *S. aureus* *E. coli*—S17 strain *S. mutans*—ATCC 25175 *A. actinomycetemcomitans*—ATCC 33384	200 μg/mL 200 μg/mL 400 μg/mL 200 μg/mL	0.3 mg/mL 0.4 mg/mL 600 μg/mL 200 μg/mL	[46,51,53,59]
	Thymol	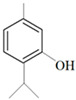	Methicillin-resistant *S. aureus* *E. coli*—S17 strain Methicillin-resistant *S. aureus*—ATCC 33591 *E. coli*—ATCC 10798 *S. mutans* ATCC 25175 *A.actinomycetemcomitans*—ATCC 33384 *E. coli*—ATCC 8739 *S. aureus*—ATCC 25923 *B. cereus*—ATCC 14579 *S. typhimurium*—ATCC 14028	200 μg/mL 200 μg/mL 200 μg/mL 200 μg/mL 200 μg/mL 100 μg/mL 7 μg/mL 7 μg/mL 7 μg/mL 3 μg/mL	0.3 mg/mL 0.3 mg/mL 200 μg/mL 400 μg/mL 400 μg/mL 200 μg/mL 0.12 mg/mL 0.12 mg/mL Bacterial growth 0.12 mg/mL	[46,51,52,53,60]
	β-caryophyllene and Squalene	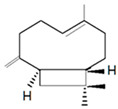 β-caryophyllene 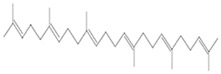 Squalene	Methicillin-resistant *S. aureus* *E. coli*—S17 strain	>4000 μg/mL >4000 μg/mL	>4.0 mg/mL >4.0 mg/mL	[53]
	Terpineol	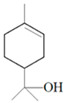	*E. coli*—ATCC 8739 *S. aureus*—ATCC 25923 *B. cereus*—ATCC 14579 *S. typhimurium*—ATCC 14028	60 μg/mL 30 μg/mL 120 μg/mL 120 μg/mL	Bacterial growth 0.12 mg/mL Bacterial growth 0.25	[52,54]
	Benzoquinone - embelin	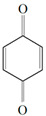 Benzoquinone 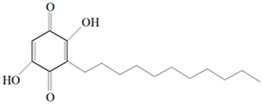 Embelin	*S. aureus*—ATCC 6538 *B. cereus*—ATCC 10876 *E. coli*—ATCC 4157 *Pseudomonas aeruginosa*—ATCC 9027	20 μg/mL 20 μg/mL 45 μg/mL 25 μg/mL	20 μg/mL 75 μg/mL 325 μg/mL 125 μg/mL	[54,61]
	Carveol	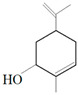	*E. coli* *S. aureus*	200 µg/mL 2000 µg/mL	1500 µg/mL 2500 µg/mL	[51]
	Citronellol	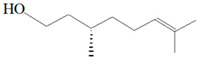	*E. coli* *S. aureus*	5 µg/mL 375 µg/mL	15 µg/mL 400 µg/mL	[51]
	Citronellal	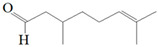	*E. coli* *S. aureus*	300 µg/mL 400 µg/mL	500 µg/mL 800 µg/mL	[51]
^a,c^ Antifungal	Nerol	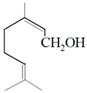	*A. niger* *Aspergillus ochraceus* *Aspergillus flavus*	300 µg/mL 300 µg/mL 200 µg/mL	300 µg/mL 500 µg/mL 200 µg/mL	[62]
	Thyme	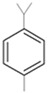 p-cymene 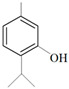 Thymol 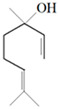 Linalool 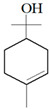 α-terpineol 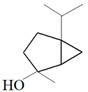 Sabinene hydrate	*A. alternata*—air-borne *C. globosum*—air-borne	250 μg/mL 250 μg/mL	0.05% (*w*/*v*) 0.05% (*w*/*v*)	[57]
^d^ Antiviral	Thymol	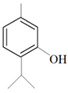	Herpes simplex virus type 1	7 µM		[63]
	Carvacrol	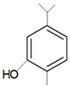	HSV-1	7 µM		[63]
	Farnesol	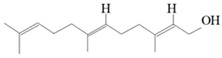	HSV-1	3.5 µg/mL		[64]
	Eugenol	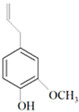	HSV-1	35 µg/mL		[64]

^a^ MIC = Minimal Inhibitory Concentration. ^b^ MBC = Minimum Bactericidal Concentration. ^c^ MFC = Minimum Fungicidal Concentration. ^d^ IC_50_ = Inhibitory Concentration 50.
